# Supplementary material for: Are carbon emissions trading and green financial instruments synergistic? -Comprehensive quantitative research based on content analysis
Source: PLoS One. 2024 Mar 7;19(3):e0298601. doi: 10.1371/journal.pone.0298601 (PMC10919601; doi:10.1371/journal.pone.0298601)
Supplement: S1 Data — (ZIP) [file pone.0298601.s001.zip › Data/地级市Z_F工作报告.zip!/║┌┴·╜¡/─╡╡ñ╜¡╩╨/└┤╫╘╣1⁄2╓┌║┼í╛┬φ┐╦╩2╛▌═°í┐_2007.docx]

各位代表：

　　现在，我受市人民政府委托，向大会作工作报告，请予审议，并请各位政协委员和列席人员提出意见。

　　一、2006年工作和过去四年发展的简要回顾

　　2006年，市政府在省委、省政府和市委正确领导下，在市人大、市政协监督支持下，认真践行“三个代表”重要思想，树立和落实科学发展观，带领全市人民拼搏进取，攻坚克难，实现了“十一五”发展的良好开局。

　　（一）经济平稳较快增长，综合实力持续增强。全市地区生产总值完成347.1亿元，同比增长12.8%。全社会固定资产投资101.2亿元，增长17.4%。规模以上工业增加值、销售收入和税金实现45.4亿元、160.5亿元和9.1亿元，分别增长12.1%、14.7%和5.3%。全口径财政收入32.6亿元，增长9.5%。城镇居民人均可支配收入和农民人均纯收入达到8035元和4686元，增长9.6%和14.7%。城乡居民储蓄存款余额394.1亿元，比年初增加25亿元。社会消费品零售总额134亿元，增长11.2%。

　　（二）改革重组和项目建设取得突破，工业经济增势明显。制药、炼油2户骨干企业当年重组、当年启动、当年见效，重组改制后的桦林等大企业和60%以上中小企业活力倍增，水泥集团重组迈出实质性步伐，牡纺等3户企业政策性破产获国家批准。23个市级重点项目有18个开工和续建，完成投资26.7亿元，其中7个工业项目完成投资7.7亿元，恒丰2万吨高档印刷型水松原纸、皓月20万头肉牛加工等项目投产，一批新项目进展顺利。全年开发新技术、新产品57项，高新技术产业产值达到62亿元。市商业银行挂牌运营。改组成立了牡丹江城市投资（集团）公司。整合后的市开发区建设步伐加快，12户投资5000万元以上的企业入区、5户开工建设。

　　（三）新农村建设实现良好开局，农业和农村发展形势喜人。投入新农村建设资金8.4亿元，新建农村水泥公路1113公里、乡镇客运站8个，新增安全饮用水人口8.6万人，涌现出海林农场、宁安小朱家村等新农村建设典型。种植业连续四年大丰收，粮食总产再创新高。四大主导产业收入占农业收入的“半壁江山”，主导产业支撑农村经济的格局初步形成，农村经济总收入实现153.8亿元，同比增长11.2%。设立了全省首家境外劳务输出服务中心，全市向域外累计转移农村劳动力14.7万人，落实国家惠农补贴资金3亿元，农民人均纯收入连续四年全省第一。绿化造林9.9万亩，治理水土流失35.1万亩，穆棱清河等3个“黑土区”、宁安响水灌区、镜泊湖水库除险加固工程列入国家农业基础设施建设投资计划。

　　（四）外经外贸持续攀升，旅游业呈现加速发展态势。全市外贸进出口总额实现62亿美元，同比增长30.2%，其中对俄贸易42.4亿美元，增长7.8%，分别占全省的48.2%和63.4%。对俄经贸产业带初具规模，发展势头强劲，年木材进口量达到776万立方米，加工能力达到350万立方米，绥--波贸易综合体一期工程落成运营，引进哈尔滨华晟国运物流公司。实施“走出去”战略，境外工业园区达到4个、企业12户。“俄罗斯年”活动丰富多彩，举办中俄边境地区经贸合作论坛等活动70多次，镜泊湖景区被联合国评为世界地质公园，首尔航班增至每周4班，韩国入境旅游者增长3倍，台湾旅游者翻了一番，全市旅游业总收入实现30亿元，同比增长16.4%，出入境旅游人数和外汇收入居全省首位。

　　（五）国内外区域合作不断深入，招商引资再结硕果。10个沪牡合作项目开工建设，到位资金6.7亿元，新签约3000吨多晶硅等22个项目，总投资48.7亿元，开展了教师培训和医疗技术协作，上海至牡丹江直飞航班复航，江海经贸发展有限公司启动运营，市城投（集团）公司与上海浦裕公司合作组建了龙裕城建开发公司，与浙江嘉兴市确定为友好市，与哈尔滨、大庆等地合作进一步深化。组织开展赴沪苏浙、香港、韩国、法国等招商活动，在韩国首尔设立全省第一家专业招商机构，“木博会”成为国家级常设展会，与韩港两地合作日益紧密，招商引资向产业集群、对俄进出口加工等领域集中，全年引进域外资金到位74.5亿元，同比增长19.3%，实际利用外资连续三年保持全省第二位，新签约亿元以上项目36个，其中16个开工、3个投产。

　　（六）保障水平进一步提高，和谐社会建设得以加强。率先在全省启动退休人员医疗保险，落实了1.7万名改制中小企业退休职工终身医疗保险，企业职工最低工资标准由305元提高到475元，城区低保金标准由120元提高到150元，改革了公务员工资制度和事业单位收入分配制度，全面实施了农村居民最低生活保障制度，半数以上农民参加新型农村合作医疗。新增就业岗位7.6万个，安置城镇失业人员6.4万人，为6850名符合条件的下岗失业人员发放社保补贴1344万元，城镇登记失业率控制在3.9%以内。投资近3000万元，整修了11条道路和人民公园、北山公园、火车站西广场。完成供热管网改造200余万平方米，大气环境优良天数达到313天，20件为民实事基本完成，爱民区社区服务中心被评为全国优秀社区服务中心。完善了安全防控和应对突发性事件综合体系，各类事故发生率平稳下降，安全度过森林防火和防汛期，加大了信访工作力度，“平安牡丹江”建设扎实推进，社会治安状况良好。

　　（七）以承办省运会为契机，各项社会事业协调发展。累计投入1.9亿元，新建北山体育中心，改造东四等7个体育场馆，体育设施投入超过建国以来各个时期的总和。省运会圆满举行，我市代表团荣获金牌总数第四名和体育道德风尚奖，城市形象和市民风貌全面展示。歌舞、戏剧等文化品牌影响扩大，群众体育、广场文化活动有声有色。整合了市区3所中学和4家卫生机构，师范学校晋升省幼专工作进入国家评审阶段，农村中小学D级危房改造全部完成，妇儿医院新楼投入使用。人口和计划生育、民族宗教、外事侨务、接待、统计、审计、民兵预备役、边防、人防、防震减灾、气象、档案、修志工作以及老区、老年人、残疾人事业取得了新成绩。

　　各位代表，市十三届人民政府任期结束。过去四年，是全市上下经受考验、奋力前行、不断求索、争取胜利的四年。四年间，全市地区生产总值年均增长9.7%，规模以上工业增加值、销售收入和利税年均增长11.9%、14.5%和11.6%，全社会固定资产投资年均增长15.2%，全口径财政收入年均增长13.3%，经济发展呈现逐步回升的良好态势。四年间，通过预算安排和争取上级财政资金，累计投入86亿元，推进了基础设施建设、江南开发、旧城改造和环境综合治理工程，城乡面貌进一步改观，道路交通条件有了根本改善，科技教育、医疗卫生和文化体育等事业取得了长足发展。四年间，社会保障体系不断完善，覆盖面不断扩大，保障能力不断增强，城镇居民人均可支配收入和农民人均纯收入分别增加2459元和1989元，人民生活质量进一步改善。四年间，市政府深入开展普法教育，始终坚持依法行政、阳光理政，主动接受市人大的依法监督、市政协的民主监督和社会各界的舆论监督，妥善办理人民代表议案建议286件、政协委员提案754件，各项工作比较好地体现了人民的意志。

　　各位代表，我们所取得的成绩来之不易，这是省委、省政府亲切关怀和市委正确领导的结果，其中更是凝聚了全市各方面的心血汗水和无私奉献。在此，我代表市人民政府，向全市各族人民，向人大代表、政协委员、各民主党派、工商联、无党派人士、人民团体和社会各界人士，向中省直单位、驻牡部队和武警官兵及外来投资者，向所有关心支持牡丹江发展的各级领导和国内外朋友，表示崇高的敬意和衷心的感谢！

　　在肯定成绩的同时，我们也深刻地认识到，全市发展中还存在一些突出矛盾和问题：一是市域经济发展不平衡，中心城市特别是市直增长速度缓慢，实力不强。二是体制与机制障碍较多，投融资体制和开发建设体制改革滞后，诚信体系不健全。三是经济结构与布局调整任务依然较重，经济一体化格局尚未形成。四是人民生活质量和生活环境亟待进一步提高，部分群众生产生活困难，人才引进难、留住难的问题比较突出。五是经济发展环境还需要进一步改善，政府部门的执行与落实能力、工作效能与管理服务水平需要进一步提高。对这些问题，我们一定高度重视，认真加以解决。

　　二、今后五年的目标任务和2007年主要工作

　　今后五年，是我市抢抓机遇、负重奋进、努力实现又好又快发展的关键时期，也是我市实施“十一五”规划、建设社会主义新农村和构建和谐社会的重要时期，人民群众期盼加快发展、渴望尽快富裕的呼声十分强烈。我们必须增强忧患意识、责任意识、赶超意识和争先意识，聚精会神搞建设，一心一意谋发展，抓住大事、解决难事、办好实事，全面开创各项工作的新局面。贯彻落实市十次党代会精神，市政府工作的总体思路是：高举邓小平理论和“三个代表”重要思想伟大旗帜，以科学发展观和构建和谐社会统领经济社会发展全局，以提高人民生活品质为出发点和落脚点，大力推进工业立市、商贸强市、旅游旺市、科教兴市战略，进一步扩大改革开放，加快社会主义新农村建设，统筹协调各项社会事业发展，保护生态环境，节约能源资源，围绕建设实力牡丹江、魅力牡丹江、和谐牡丹江，倾力打造现代加工业基地、东北亚区域商贸中心、全国著名旅游休闲度假胜地、黑龙江东南部区域中心和全省最适宜人居创业城市，不断完善城市功能，努力实现追赶型、跨越式发展。

　　按照经济总量、财政收入、人民生活水平“三个同步提升”和科学发展、和谐发展的总体要求，今后五年全市经济社会发展预期目标为：地区生产总值年均增长11%以上，突破600亿元，人均地区生产总值超过2万元；全口径财政收入年均增长10%左右，突破50亿元，其中，市本级和市直财政收入年均增长8%左右，分别达到20亿元和12亿元；城镇居民人均可支配收入年均增长8%，达到1.1万元；农民人均纯收入年均增长7%，达到6500元；社会消费品零售总额年均增长9%，达到220亿元；城镇登记失业率控制在5%以内，市区普及高中教育，社区卫生服务人口覆盖率达到98%以上；万元地区生产总值能耗年均下降4%以上；中心城区规划建设面积达到100平方公里，总人口达到100万人。在确保完成预期增长目标的基础上，我们要自加压力，做到好中求快、更好更快，力争五年内成为全省工业大市、财政强市和人均收入最高地区之一，达到全省地市上游水平，全国同等城市中游水平。

　　千里之行，始于足下。今年，我们要按照市委的统一部署，坚持一手抓当前、一手抓长远，既要敢闯敢干，更要讲求实效；既要立竿见影，更要增强后劲；既要注重发展，更要关注民生；既要发展速度，更要发展质量，务求在“全面提升一个龙头地位、努力实现五大发展瓶颈突破、着力抓好八项重点工作”上取得实效，确保全面完成或超额完成各项任务，全市地区生产总值增长12%，全社会固定资产投资增长25%，规模以上工业增加值、销售收入和税金增长13%、15%和11%，农村经济总收入增长6%以上，外贸进出口总额增长20%，全口径财政收入增长8%，其中市直财政收入增长6%，城镇居民人均可支配收入增长10%，农民人均纯收入增长7%以上。

　　全面提升一个龙头地位：

　　大力推进哈--牡--绥东对俄经贸产业带和陆海联运大通道建设。实施口岸牵动、哈--牡--绥东沿线联动，加快打造省内新的经济增长极。明晰产业带发展战略。近期着力推进“牡海宁”、“绥（东）穆（林）”两极互动，把木业作为产业带发展的切入点、支撑点和增长点，发挥优势，突出特色，迅速形成规模效益。中远期实现“双向延伸”，向内建设与哈大齐工业走廊比翼齐飞的全省开放带、经济带、旅游带、文明带；向外连接俄罗斯乌苏里斯克、海参崴等城市，发展跨国经贸科技合作区。整体推进产业带建设。坚持整合规划、共享品牌、协调政策、错位发展，开发建设产业带沿线“一区”、“六园”。依托木材、纸浆、石油等进口资源，突出抓好以市开发区、绥--绥工业园、东宁浙江工业园和海林木业城等园区为重点的木业产业平台，加快建设以绥芬河、东宁为前沿，绥阳、下城子、市区和海林为腹地的进口加工基地，壮大牡丹江、绥芬河、东宁三个省级对俄出口加工基地，优先发展俄罗斯需求较旺的轻纺、机电、食品、建材及装饰材料等产业。今年，产业带新开工5000万元以上项目29个，其中市开发区开工10个。提升大通道功能。立足产业带、服务哈大齐，确保“借港出海”、出境不出口陆海联运大通道正式运营，开通黑龙江省新的出海口。新建绥芬河铁路口岸客运候车大厅，绥芬河国际客运站和铁路电子口岸投入使用，启用绥芬河铁路液体换装线，建设绥芬河至下城子铁路复线，争取东宁口岸界河桥、东--波互贸区外交换文，推进东--乌国际铁路建设实质性运作，实施牡丹江机场改造工程，着手制定新机场建设规划。大力发展物流园区和专业物流中心，重点抓好华晟国运物流、绥芬河国际物流、海林华运物流和下城子物流中转集散中心建设。

　　努力实现五大发展瓶颈突破：

　　（一）在盘活存量、导入增量、扩张总量上实现突破。围绕当期增效益、长远添后劲，全力保持固定资产投资合理较快增长。加快企业改制重组。确保水泥集团重组成功，完成牡纺等3户企业政策性破产，继续争取桦林集团等企业政策性破产获国家批准，盘活绿津实业、牡康等企业闲置资产，启动厂办大集体企业改制试点，推动改制企业解决遗留问题、不断靠大联强。促进项目批量落地。确定投资规模5000万元以上的市级重点项目54个，总投资466亿元，其中工业项目23个。今年计划投资41亿元，确保4万吨醋酸乙烯改扩建等13个项目竣工投产，续建460万套子午胎等16个项目，“双16万吨”烧碱和树脂等10个项目开工建设，3000吨多晶硅等7个项目争取开工，加快3万吨海绵钛等8个项目前期运作。全力抓好“上争外引”。做实项目谋划和储备工作，积极落实项目配套资金和建设条件，为向上争取和招商引资打好基础。把引进重大项目和战略投资者作为主攻方向，强化政府搭台、企业主体的双重作用，巩固韩国、香港和长三角、珠三角招商市场，积极拓展欧美等招商市场，开展多种形式的务实招商，抓好签约项目跟踪问效，扩大招商成果。全年引进亿元以上项目22个，域外资金到位85亿元，实际利用外资7400万美元，同比分别增长15%和18%。

　　（二）在资源整合、区域联动、整体发展上实现突破。强化大牡丹江观念，坚持行政手段与市场手段相结合，加快区域经济一体化进程。加大重大资源整合开发力度。成立全市重大资源开发项目专题推进组，对市域内水能、风能、矿产、林木等资源整体规划、统一运作、合理开发，降低运作成本，提高资源开发利用率和资源性项目招商落地率。创新利益联接机制。以效益为纽带，制定区域内资源共享、政策共享、平台共享、利益共享的措施办法，打破区域、条块界限，促进市县区之间、地方与农垦和森工等中省直单位之间的合作，构筑区域经济整体优势。按照重心下移、责权利相一致原则，明确事权划分，建立“两级政府、三级管理、四级网络”的城市管理体制。放大园区集聚效应。坚持项目园区化、园区专业化，制定园区产业发展指导目录，进一步规范园区发展，优化园区布局，完善各园区功能定位，构建以市开发区为龙头的国家、省、市三级园区体系，积极发展以区中园、园中园为主要形式的“飞地经济”。迅速壮大县区经济。深入贯彻落实省委《关于进一步加快县域经济发展的决定》，支持绥芬河、东宁口岸县（市）率先发展，鼓励绥芬河加快“北方深圳”建设，扶持内陆县（市）发挥比较优势，发展特色产业，围绕全国“百强”、全省“十强”目标，升位争先。各城区也要因地制宜，走主导产业立区、招商引资兴区的道路，扩大城区经济规模，增强发展活力与实力。

　　（三）在完善投融资和城市开发体制、提升资本运作水平上实现突破。以创新体制机制为前提，以市场化运作为核心，畅通融资渠道，有效破解资金瓶颈。构筑安全有效的投融资平台。深化投融资管理体制改革，加大资源、资产、资本“三资”注入力度，做大投融资平台。严格履行借、用、管、还责任，强化对项目运作、资本金及投融资的调控，理顺开发建设体制，加快城市开发建设进程，提高建设资金使用效率。建立多元化融资机制。加强信用建设，营造良好的金融生态环境，巩固与开发银行和各商业银行的合作关系。深化农村信用社改革，扶持城市商业银行发展，壮大地方金融机构实力。积极引进外资银行和国内股份制银行分支机构以及保险、信托、证券、基金等其他金融机构，发行企业债券，活跃民间资本，扶持有条件的企业上市融资。全方位搞好城市经营。利用市城投（集团）公司平台，坚持储备统一渠道、开发统一运作、收益统一监管的“三位一体”模式，对城市基础设施和公益类、资源性项目以及政府承贷项目进行整体包装和市场化运作，提高成功率。

　　（四）在培植壮大财源、建立健全公共财政体制上实现突破。完善“生财、聚财、理财、用财”措施，逐步建立以民生为本、有利于各项事业协调发展的公共服务均等化财政分配体制，为构建和谐社会奠定坚实的财力基础。大力培植各类财源。加速骨干企业改制重组步伐，清理规范财税政策，统一政策标准，公平发展环境，整合各类财源建设资金集中打捆使用，做大存量财源。通过招商引资、项目建设，发展“总部经济”、“结算经济”和“参股经济”，培植新生财源。积极争取国家和省政策性资金及转移支付资金，扩充政策性和体制性财源。完善市、县（市）、区财源项目税收分享办法，扶持共享财源。坚持依法从严治税，强化重点税源监控，建立市直税收监管机构和全市税源管理信息共享平台，防止税源流失，稳固既得财源。加强非税收入管理，逐步将国有资本收益、国有资源有偿使用和特许经营等收入纳入管理范围，拓展非税财源。深化财政管理体制改革。巩固部门预算、综合预算、国库集中收付、政府采购制度等改革成果，全面推进政府收支分类改革，实施国有资本经营预算管理制度，推行机关后勤服务社会化、供热货币化改革，探索公车改革，认真做好统一内外资企业所得税准备工作。按照省里要求，年底前完成事业单位综合配套改革。严格财政资金监督管理。完善监督机制，对财政资金进行全程监督，发挥财政投资评审作用，提高投资评审质量，确保财政资金运行规范安全。做好项目绩效综合评价工作，提高财政投资和支出效益。出台债务管理办法，规范政府举债、使用、偿还和担保行为，完善政府债务风险预警和准备金制度，保证到期债务及时偿还。提高公共财政保障能力。优化财政支出结构，加大新农村建设、就业再就业和社会保障、社会事业发展和生态环境保护等投入力度。认真落实党政机关、事业单位、离退休人员工资改革和套改、发放地区津贴等政策措施。完善住房货币化补贴资金发放制度，对符合条件的职工及时发放住房补贴。推行廉租住房保障制度，进一步改善城镇低收入困难群众住房条件。

　　（五）在经济结构和布局调整、增强发展活力上实现突破。高度重视经济社会发展规划，坚持工业化、产业化与城市化互动，坚持用规划推动建设和管理，提高经济结构层次，优化生产力空间布局，培育壮大新的经济增长点。合理确定产业布局。依据城市功能定位，打破行政区域界限，加快建设以进出口加工基地和商贸物流中心为主的中部板块，以精品旅游为主的西部板块，以森林及矿产资源加工为主的北部板块，以生态及现代农业加工为主的南部板块。围绕两大城市组团布局，铸就城市精神，塑造城市品牌，强化城市功能，提升城市能级。以实现“双百”为目标，拓展城市发展空间，加快户籍制度改革，优先做大中心城市，提高人口承载和产业集聚能力；加快打造“绥东绥”城镇化组团，配套发展卫星城，协调发展小城镇，有序发展新农村，形成外向型、加工型、旅游型、商贸型特色小城镇体系。调整优化工业结构。改造传统优势产业，整合提升大庆路工业园区，再造牡丹江工业新优势。围绕做大森林工业、汽车配套、石油化工、新型材料、医药、能源六大支柱产业，推进产业资源整合，延伸产业链。巩固扩大恒丰、大宇等纸业企业规模和市场份额；扶持桦林佳通、富通等汽车配套企业，提高整体配套能力和产业层次；通过重组、改造和上新项目等途径，形成以东北化工、东北高新等企业为支撑的化工产业；以高科生化为龙头，开发整合“北药”资源，增强药业企业整体竞争力；加快发展木业产业，提高木材深加工水平；以多晶硅项目为牵动，促进圣戈班、顺达电石等企业提档升级。围绕食品工业、农业资源深加工、进出口加工、高新技术产业、绿色能源和循环经济，大力发展新型工业。壮大旅游和会展经济。以“对俄大通道、生态牡丹江”为主题，精心打造镜泊湖、林海雪原和百年口岸三大品牌，扩大镜泊湖世界地质公园的影响，争取镜泊湖成为国家5A级景区，创办“镜泊湖之夏”旅游节，丰富冬季冰雪特色旅游项目，大力兴办乡村旅游。强化旅游市场营销，拓展旅游客源市场，规范提高旅游业服务水平，全年旅游人数和旅游业总收入实现两位数增长。推进“十大旅游精品工程”建设，深度开发特色旅游商品。依托产业带、立足牡丹江、辐射黑龙江、面向东北亚，精心承办第三届中国（牡丹江）木博会，办好首届牡丹江雪堡经贸旅游洽谈会、中国（牡丹江）农业产业经济高峰论坛暨特色农产品博览会、中国（东宁）黑木耳节和绥芬河元宵灯节等展会活动，规划筹建牡丹江国际会展中心。优化提升城市商贸商务功能。坚持功能错位、突出特色，调整商业网点和布局，提升发展中心城区零售商业，规范发展社区商业和地区商业副中心，加快发展交通枢纽型商业，创新发展批发贸易业，培育发展生产型服务业。规划建设东安中央商业商务区、西安民族商业区、阳明汽贸服务区、爱民大学城服务区和江南新城现代服务区。积极发展金融服务业、现代房地产业、第三方物流、行业协会和中介组织，培育休闲、健身、文化等新型消费服务业，不断改善城乡消费环境，促进居民消费升级。扶持非公有制经济发展。放大工业发展专项资金调节作用，建立中小企业互助基金，积极争取国家和省科技专项资金、贷款贴息，发展“两台一会”，增强中小企业融资能力。实施“中小企业成长工程”，辟建江南中小企业创业园，扶持劳动、技术和资本密集型等民营企业发展，形成专精特新、机制灵活的中小企业群体。全市非公有制企业实缴税金增长12%，市区20户“小巨人”企业销售收入增长20%，新增30户以上规模工业企业。

　　着力抓好八项重点工作：

　　（一）以对俄、对韩合作“双升级”为牵动，全方位扩大对外开放合作。充分利用国际国内两个市场、两种资源，在推进对俄经贸科技合作的同时，加速对韩合作战略升级，扩大与其他周边国家和国内城市跨区域合作，增强市域经济发展的外部动力。推进对内对外合作。对内围绕以上海为中心的长江三角洲和以哈尔滨为中心的省内城市，对外以俄罗斯、韩国为重点，加快建设跨区域合作的前导区、示范区。大力度推进沪牡合作，进一步创新合作模式，拓展合作领域，全方位对接项目，努力在经济、社会等各领域合作上取得新突破。加强与省城及省内各地市合作，积极参与黑龙江东部煤电化基地建设，建立与周边城市的战略合作伙伴关系。完善对俄合作规划，着手制定对韩合作规划，以进出口加工为导向，按照“中中外”、“中外中”模式，办好境外工业园，辟建绥芬河远东工业园和乌苏里斯克康吉经贸合作区，联合境内外企业和引导本地企业与俄方开展资源能源开发及种养业、旅游、房地产开发、建筑工程承包、金融、高新技术等领域的合作。办好中俄信息产业园，探索引进韩国企业建设韩国工业园。发展对外贸易。以专业化、现代化为方向，引导绥--波、东--波互贸区错位经营，完善绥芬河青云、迎泽和市区朝鲜民族商场等专业市场功能，促进哈巴罗夫斯克亚洲国际商贸城投入运营。转变贸易增长方式，提高地产品、农产品、机电产品和高科技产品出口规模，扩大能源、原材料等进口规模，积极培育省级出口品牌。巩固传统市场，拓展欧美等发达国家地区市场，开拓南美和非洲市场，构建贸易伙伴多元化格局。扩大对外交流。抓住“俄罗斯·中国年”和“2007中韩交流年”的有利契机，在俄罗斯远东地区和韩国首尔、坡州等城市全面宣传展示牡丹江，推动对俄对韩经贸、科教、文体等各领域交流合作，掀起“牡丹江热”。落实与俄罗斯乌苏里斯克、韩国坡州等友好城市定期互访机制，促进友好城市向合作城市发展。

　　（二）大力发展现代农业，奠定新农村建设的物质基础。以产业化经营牵动现代农业发展，推动农业不断增效、农村加快发展、农民持续增收。培育现代农业产业体系。优化农业产业结构和生产布局，做大做强四大主导产业和特色养殖、园艺等产业，引导“一村一品”向优势产业集聚，促进农业向产业化、规模化、区域化方向发展，四大主导产业收入占农业总收入的比重达到55%以上。发展农产品精深加工，壮大皓月、鑫鹏、三通、草原兴发等加工型龙头企业，再培育一批省和国家知名农产品品牌。抓好10万头无公害肉牛、10亿袋（块）绿色有机食用菌、10万亩对俄绿色果菜、10万头无公害生猪四大产业基地建设，创建穆棱肉牛、东宁黑木耳、宁安绿色水稻三个国家级农业标准化示范区。提高现代农业综合生产能力。实施科技入户工程，全面推进农业科研机构、大专院校、科技推广部门、农垦与市县乡开展农业科技共建，创新农技推广机制，大力推广工厂化农业生产、测土配方施肥、新型能源等新技术。推进土地适度规模经营，土地流转达到100万亩。提高现代农业装备水平，田间作业综合机械化程度达到78.5%。加强农业基础设施建设，推进国家级响水大型灌区、镜泊湖水库除险加固、牡丹江左右岸防洪工程和“黑土区”水土流失综合防治等工程，治理水土流失面积26万亩。完善现代农业服务体系。健全以批发市场为中心、集贸市场和零售经营为基础的农产品流通网络，发展壮大绥阳黑木耳、双合果菜、东宁哈达等一批年交易额超亿元的专业市场，建设黑龙江省对俄农业科技合作园区，加快农产品进军国际市场步伐，地产农产品出口额达到3.5亿元。引导扶持农民和龙头企业兴办各类专业协会、合作社、股份公司等合作组织，提高农民组织化程度。实施农村实用人才培养工程，积极开展装备之冬、阳光培训、百万中专生培训等工作，培训农村实用人才30万人次，新转移农村劳动力1.8万人。在发展生产的同时，继续改善农村生产生活条件，抓好新农村试点，推进康居乡镇、康居新村和少数民族乡村建设。

　　（三）实施开发与改造互动，提升城市建设管理水平。坚持国家环保模范城、卫生城和山水园林城“三城同创”，改造老城、开发江南、集聚产业、连片发展，增强城市承载能力。加强城市基础设施建设。江南新城区兴隆组团基础设施工程实现“水通路畅成环闭合”，市开发区北区今年全部完成规划范围内基础设施建设，市民广场、市一中新校区等项目投入使用，推进东四跨江桥二期工程建设，力争开工建设远程物流五星级宾馆等一批新项目。建设城市规划展示馆、博物馆、青少年宫和革命烈士馆等公共设施，加快以桥北及各城区城乡结合部为重点的棚户区整体搬迁改造和区域开发。整修城区道路，打造光华街样板街路，抓好城区照明续建工程，力争实现主城区大街小巷全部亮灯，加强基础设施亮化、楼体亮化、牌匾广告亮化，营造城市夜色景观。健全城乡交通网络。围绕增强区域性交通枢纽功能、构建现代城市交通网络，开工建设牡绥高速公路、鹤大公路牡丹江至宁安段、鸡图公路老黑山至省界段、鸡讷公路林口至大罗密段，确保年内实现绥满公路虎峰岭至海林界段、鹤大公路宁安至杏山段及杏山至省界段竣工通车。综合整治城市环境。抓好“三山、三花、两树”绿化工程，打造沿江、沿山景观带，开展市花、市树评选活动。实施工业污染、低空面源污染和大气污染治理攻坚战，推进小锅炉并网，大力推广民用型煤，大气环境优良天数保持在300天以上。启动市污水处理厂运行，建设海林和宁安污水处理厂，争取林海水库供水工程开工，提高居民饮用水安全度。认真落实城市属地化管理权限，完善城管执法目标化管理和公安、城管联动机制，集中开展以交通秩序、户外广告、占道经营、违章建筑为重点的市容市貌综合整治活动。完善物业管理企业、业主委员会、居民委员会和公安派出所“四位一体”管理体制，提高物业管理水平。改造城市生活垃圾收运系统，推行分类收集管理，做好日常保洁工作，美化城市环境。

　　（四）加快创新型城市建设，推动经济社会可持续发展。坚持把增强自主创新能力作为转变经济增长方式的中心环节来抓，靠创新构筑竞争优势。发挥人才保障作用。完善人才工作政策措施，调动现有人才积极性，加大“招商引智”力度，培养引进一批科技拔尖人才和领军人才。充分发挥“外脑”作用，借助外地人才的力量，帮助我市提高创新能力。深入实施职工素质工程，广泛开展科普活动，不断提高劳动者技能。鼓励各领域和行业开展创新活动，营造尊重劳动、尊重知识、尊重人才、尊重创造，有利于各类人才脱颖而出的社会氛围。强化科技支撑能力。围绕电子信息、生物技术与医药、先进制造业与化工、新能源和节能环保四大领域，重点抓好特种材料和石油钻采装备两个国家“火炬计划”产业基地建设，培育恒丰、金刚钻碳化硼等一批高新技术企业集团，支持企业与科研院所开展技术联盟，不断研发新技术、新产品，大力培育企业品牌，加强知识产权保护。全年实施省级以上重点科技攻关项目30项、高新技术产业化项目20项，新增高新技术产品10项、省级以上高新技术企业5家，高新技术产业产值突破70亿元。加快发展循环经济。全面组织实施循环经济园区试点方案，围绕煤化工、造纸、热电联产等产业谋划和申报项目，积极争取国家和省支持，确保一批项目开工建设。充分利用试点政策，完成5个省级和30个市级试点企业废水治理和综合利用项目建设。按照“减量化、再利用、资源化”原则，制定全市循环经济发展规划，推进循环经济向宽领域、深层次发展。

　　（五）统筹发展社会事业，增强社会发展活力。以满足人民群众公共服务需求为重点，优化社会资源，促进社会进步，为和谐社会建设提供有力支撑。优先发展教育事业。全面落实农村义务教育经费保障机制，完善教师援助制度，建好农村寄宿制学校，不断提升农村教育资源配置水平，推动城乡教育均衡发展。在此基础上，以强化城市服务功能、建设区域性教育中心为目标，推行“四个一流”示范工程，组建幼儿教育中心，进一步改善各类学校办学条件，巩固提高省级示范高中办学水平，推进市朝中和穆棱一中争创省级示范高中，鼓励社会力量办学，积极引进国内各层次名校创办分校，做大做强牡丹江大学，支持在牡高校发展，规划建设大学城，创办教育产业园。大力发展职业教育，支持市高级技工学校申办技师学院，加快建设一批职业教育实训基地，满足社会就业创业需求。强化公共卫生保障。加强农村卫生院建设，全面推行农村新型合作医疗制度。调整区域卫生机构设置，扩大社区卫生服务覆盖面，降低群众医药消费支出，为群众提供良好的基本医疗服务。深化与国内知名医院交流合作，发展特色专科医院，培育名医名院。完善公共卫生体系，提高公民卫生保健意识，有效预防各类传染病。繁荣各项社会事业。加快发展文化事业和文化产业，活跃广场文化、社区文化等城乡群众性文化活动，壮大戏剧、歌舞等演出团体，创作文艺精品，开展历史遗迹保护和非物质文化遗产普查，完善传媒集团文化改革试点，抓好广播电视数字化工程建设。推进北山体育中心等场馆市场化经营，为开展全民健身活动创造条件。广泛开展拥军优属、拥政爱民活动，争创全国双拥模范城“五连冠”，抓好第二次农业普查，做好人口和计划生育、民族宗教、外事侨务、接待、审计、民兵预备役、边防、人防、防震减灾、气象、档案、修志等工作和老区、老年人、残疾人等事业。

　　（六）注重解决民生问题，保持社会和谐稳定。想群众之所想，急群众之所急，切实维护好、实现好、发展好人民群众的切身利益。扩大就业再就业。建立健全劳动力市场服务体系、劳动保障工作平台体系、再就业技能培训体系和下岗失业人员保障机制，重点解决好“4050”人员和“零就业”家庭就业问题，新增城镇就业6万人，实现再就业5万人，做好大学毕业生就业和复员转业军人安置工作，加强劳动监管和劳动仲裁工作，构建和谐的劳动关系。增强社会保障能力。大力推进以社会保险和城市低保、农村低保制度为基础、专项救助相衔接、临时救助为补充、优惠政策和社会互助相配套的社会保障及救助体系建设，发展慈善事业，加强福利设施建设，加大对社会困难群体帮扶力度，引导社会力量兴办扶老、救孤、助残、济困等社会福利机构。落实《妇女儿童发展规划纲要》，保障妇女儿童合法权益。完善信访体制机制。组建市信访工作委员会、县（市）区群众工作局和乡镇（社区）信访调处中心，搭建三级信访工作平台。成立市联合接待中心和受理中心，设立协调督查办公室。构建人民调解、司法调解和行政调解“三位一体”工作体系，健全完善社会矛盾和纠纷调解机制。开展“信访攻坚行动”，集中解决好群众反映强烈的信访问题，努力做到解决一个问题，形成一项制度，化解一批矛盾。推进“平安牡丹江”建设。落实安全生产责任制和一票否决制，健全安全生产监管网络，强化生产企业、道路交通、建筑工地及各类危险源、事故源的监管，严防关停小煤矿反弹，坚决防止重特大安全事故发生。加强应急设施建设，完善突发事件应急体系，提高应急处置和公共安全保障能力。健全社会治安网格化管理体系，建立刚性务实的警力下沉机制，加强对重点部位和重点人群的管理，严厉打击各种犯罪行为，增强市民的安全感。举办首届“社区节”等活动，促进邻里和睦、社区和谐，打牢稳定和谐基础。

　　（七）优化经济发展环境，打造投资创业热土。从影响环境的关键环节和突出问题入手，标本兼治，重在治本，为全民创业、招商引资、加快发展扫清障碍、创造条件。提高行政审批效率。对所有投资项目实行领办代办、限时办结、超时默认制度，重大项目实施规划、立项、土地、环保“四审”联动和市级领导代办制，加快项目落地。加强行政审批服务中心建设，制定更加有力的约束措施，彻底解决体外循环和效率不高问题，对部分常规审批项目实行告知制。规范收费检查行为。发挥市优化办的作用，严格审批针对企业进行的检查活动，建立集中定期收费制度，未经市优化办许可一律不得到企业检查收费。开通“市长环境热线”，从严惩处乱收费、乱罚款、乱摊派等行为，全力为企业发展保驾护航。深化行政审批制度改革，最大限度地减少和规范审批事项，实行“阳光收费”。强化跟踪服务。对引进企业和落地项目，实行“保姆式”、“全天候”服务，及时解决项目建设和企业生产经营中遇到的各种困难，多为企业解忧，不给企业添乱。完善“双评”措施，企业、部门、群众和中介机构共同参与行风政风监督测评，促进市直及中省直部门积极主动地为企业服务。优化信用环境。深入开展“诚信政府、诚信企业、诚信社会、诚信市民”创建活动，加快建设社会信用体系，建立信用评价、守信激励和失信惩戒机制，着力打造诚信牡丹江。营造亲商创业氛围。视投资者为“上帝”，视纳税人为“衣食父母”，建立政府与本地企业和外商联系制度，定期开展联谊活动，邀请企业经营者参加政府的重要会议，及时听取企业对政府的意见和建议。把创业作为富民的根本之策，完善扶持政策，放宽前置条件，鼓励群众兴家业、能人办企业、干部创事业。

　　（八）全面加强政府自身建设，为加快发展提供有力保障。致力于建设责任政府、服务政府、法治政府，以求真务实、真抓实干的工作作风取信于民，以立言立行、雷厉风行的实际行动取信于民，以勤政为民、廉洁自律的政德政风取信于民，以经济实力增强、人民生活富裕的发展业绩取信于民。牢固树立以人为本的施政理念。坚持发展依靠人民，最大限度地调动广大群众投身发展的积极性、主动性，凝聚全市各方面力量，推动牡丹江跨越式发展。坚持发展为了人民，做任何决策都要以群众满意不满意、高兴不高兴、答应不答应为前提，干任何事情都要以民为重、以民为上、以民为先；坚持发展惠及人民，把雪中送炭与锦上添花有机结合起来，每年集中办好一批利民惠民实事，下大力气解决好群众最关心、最直接、最现实的利益问题，让全市人民充分享受改革发展的成果。坚持依法行政、开展绩效评估。把依法用权、依法办事贯穿于政府工作的各个方面、各个环节，全面落实行政执法责任制和行政执法过错责任追究制，做到严格执法、公正执法、文明执法、高效执法。自觉接受人大依法监督、政协民主监督和社会各界的舆论监督，认真办理人大议案、建议和政协提案，全面推行政务公开，确保政府各项决策民主、科学、规范。抓好效能监察和绩效评估，认真监督检查政府机关和公务员履行职责的情况，严肃查处推诿扯皮、不负责、不作为的行为，从严治政，不断增强政府的执行力、落实力、公信力。提升社会管理和公共服务水平。按照转变职能、权责一致、强化服务、改进管理、提高效能的要求，加快推进政企分开、政资分开、政事分开、政府与中介组织分开，把政府工作的重心从单纯抓经济发展、单纯追求经济增长速度真正转移到经济调节、市场监管、社会管理和公共服务上来。围绕增强社会文明度、经济富裕度、环境优美度、资源承载度、生活便宜度和公共安全度，创新管理和服务模式，拓宽服务领域，改进服务质量，不断提高人民群众对政府的满意程度。加强公务员队伍建设。深入开展学习型机关、学习型干部队伍创建活动，提高公务员业务素质，增强各级政府领导干部驾驭工作全局、领导科学发展、推动改革创新、处理复杂问题的能力和水平。强化工作目标管理考核，激励先进、鞭策后进。建立完善廉政建设责任制、行政监察、财政监督和审计监督等制度，坚决遏制各种不正之风，严肃查处违反行政纪律和侵害群众利益的案件，坚决做到用制度管权、靠制度管人、按制度办事。

　　各位代表！加快发展、造福人民，是新一届政府必须认真履行的神圣职责。让我们在市委的坚强领导下，凝聚275万牡丹江各族人民的智慧和力量，解放思想，创新创业，锐意进取，奋发图强，竭尽全力地把我们的宏伟蓝图变为美好现实，共同拥抱牡丹江全面振兴的美好春天！

　　我们的目的一定要达到，我们的目的一定能够达到！
